# Clinical Cases Treated in the Surgical Wards of the Mercy Hospital

**Published:** 1857-04

**Authors:** J. W. Freer

**Affiliations:** Attending Surgeon


ARTICLE IV.—*Clinical Cases treated in the Surgical Wards of the Mercy Hospital, during the Session of Rush Medical College, for* 1856–'7. J. W. FREER, M.D. Attending Surgeon.

Michael Barren, admitted Oct. 20th, with simple fracture of the upper third of femur. Discharged Dec. 15th.

Jas. Gillman, admitted Jan. 6th, with simple fracture of the upper third of femur. Improving, but still in Hospital.

FRACTURES OF LEG.

Jno. H. Poor, admitted Dec. 15th, with fracture of the lower third of tibia. This case, owing to the limb having been badly frozen, is still under treatment.

Luke Harny, admitted Jan. 24th, with simple fracture of leg, of nineteen days standing. The history of this case may perhaps interest the advocates of the starch-bandage in cases of fracture.

The patient received an injury, Jan. 5th, which resulted in a fracture of both bones of the right leg, with considerable contusion of the soft parts. His physician directed the limb to be placed on a pillow, and cold to be applied by means of cloths dipped in cold water. The above treatment was continued for one week; the tissues in the meantime having assumed a healthy appearance, a starch-bandage was applied.

The patient at this time inquired of his physician, 'how long he should remain in bed,' and was informed that it would probably require six weeks for the bones to unite. The patient thinking that the Doctor's object in keeping him in bed so long was merely to make a fee, became highly indignant, and dismissed him, informing him 'that he, [the patient], knew a natural bone-setter who could cure him in two weeks; that he would not submit to being confined to his bed for six weeks; and that he would get up as soon as the bandage dried.' How many days he remained in the recumbent posture after the bandage was applied, we are unable to say, but that he walked a half a mile on the 24th of January we are positive.

At the time of his admission (January 24), the bones seemed to be in perfect apposition and tolerably well united. The bandage being loose, a new one was ordered, and the patient directed to remain quiet; this, however, he obstinately refused to do. After stating to him the danger he incurred, &c. he was permitted to use his own discretion, in regard to walking about. Seeing that we were not disposed to compel him to remain still, he walked about the wards every day, and most of the time without crutches.

On the 9th Feb. the bones were found, on examination, to be so perfectly united, as to no longer require a bandage, and the patient was discharged.

Pat Murphy, admitted Jan. 17th, with compound fracture of the upper third of tibia.

James Welch, admitted Feb. 17th, with fracture of the upper third of tibia, and head of fibula.

The two last cases are still under treatment, and doing as well as the nature of the injuries will admit.

Jas. Carbine, admitted Jan. 24th, with fracture of outer third of clavicle. Discharged Feb. 16th.

### EXTIRPATION OF TESTICLE.

Andrew Keenan, admitted Nov. 23d, with hypertrophy of right testicle.

The history of this case, so far as we were able to learn, was as follows:—Two years previous to his admission into the Hospital, he contracted gonorrhœa, which subsided in two weeks under the internal use of balsam of copaiba and injections of nitrate of silver. Three weeks after the subsidence of the gonorrhœal discharge, he noticed a slight swelling in the right testicle, with some tenderness. He immediately consulted a physician, who compressed the testicle by means of adhesive straps. Finding, however, at the end of two weeks, that no impression whatever had been made upon the condition of the testicle, notwithstanding the straps had been applied frequent enough to keep up an uniform degree of pressure, he abandoned their use. From that time until within six months of his entrance into the Hospital, he had been subjected to no treatment. Finding that the testicle had already reached such a magnitude as to be inconvenient from that cause alone, he consulted a surgeon with a view of having it removed. This the surgeon declined doing, but directed him to use iodide of potassa, in eight grain doses, three times a-day, and to apply a liniment composed of equal parts of olive oil and tinct. iodine as a local application. The patient followed the above prescriptions for three months, but finding the testicle still increasing in size, abandoned their further use.

At the time of his admission, the testicle, upon examination, was found to be very much indurated, highly sensitive to the touch, and to measure nine inches in its greatest circumference.

The patient being very solicitous to have the testicle removed without delay, the usual operation for extirpation was performed on the day of his admission. Nothing unusual occurred during or subsequent to the operation. The wound healed kindly, and the patient left the hospital quite well on the 26th of Dec.

The tunica vaginalis was found adherent to the body of the testicle in several places, and, on laying the testicle open, its structure appeared much altered. It differed from the simple sarcocele, there being considerable fatty deposit; and, in one place, about the size of a pea, the texture was reduced to a semi-fluid consistence, somewhat resembling the softening of tuberculous deposits: and yet the whole organ had not the irregular, or knotted appearance of a tuberculous testicle, as described by Dr. Smith.

### AMPUTATIONS.

There were, also, two cases of amputation of the thigh, one of which was for disease involving the cartilages of the knee-joint; the other, for a malignant tumor involving nearly the whole leg. A full account, however, of the two latter cases will be given in the next number of the *Journal.*

